# Analysis of Flavor Characteristics in Lemon-Fermented Fruit Wines from Three Distinct Varieties Using Odor Activity Values Combined with Multivariate Statistical Approaches

**DOI:** 10.3390/foods15162922

**Published:** 2026-08-20

**Authors:** Sen Huang, Zhihong Lu, Jiying Zhang, Yu Zhang, Zegang Li, Wanfu Yao, Yujiao Cheng

**Affiliations:** 1 Citrus Research Institute, Southwest University, Chongqing 400712, China; 2 National Citrus Engineering Research Center, Chongqing 400712, China; 3 College of Food Science, Southwest University, Chongqing 400712, China; 4 Pingshan County Fengshuo Agricultural Investment Co., Ltd., Yibin 645350, China; 5 Yuxi Academy of Agricultural Sciences, Yuxi 653100, China; 6 Key Laboratory of Quality and Safety Control of Citrus Fruits, Ministry of Agriculture and Rural Affairs, Chongqing 400712, China

**Keywords:** lemon-fermented fruit wine, GC–MS, MDGC–MS, multivariate statistical analysis, flavor characteristics

## Abstract

Fruit wines fermented from citrus fruits are highly popular worldwide. The flavor profiles of fruit wines (ELFW, SLFW, PLFW) fermented from three different varieties of lemons (Eureka lemon, ELFW; Sweet lemon, SLFW; and Perfume lemon, PLFW) were investigated by odor activity values combined with multivariate statistical analysis. Volatile compounds in fruit wines were qualitatively and quantitatively analyzed using solid-phase microextraction (SPME) coupled with gas chromatography–mass spectrometry (GC–MS) and multidimensional GC–MS (MDGC–MS). A total of 85 volatile compounds were identified across the samples, with ELFW containing the highest number (67), followed by SLFW (65) and PLFW (58). Alcohols (51.25–73.50%) dominated the volatiles, followed by hydrocarbons and esters. α-Terpineol, 4-terpineol, and d-limonene dominated ELFW, SLFW, and PLFW respectively. Forty-six characteristic flavor compounds were screened, and fenchol showed the highest OAVs. Using OPLS-DA combined with variable importance in projection (VIP), 17 key flavor markers were identified to distinguish flavor difference among three lemon cultivar-based fermented fruit wines.

## 1. Introduction

Lemon (*Citrus limon* (L.) Burm. f.) is one of the most valuable and distinctive flavor sources in the Citrus genus. Endowed with abundant vitamin C, citric acid, minerals, and flavonoids, lemons play pivotal roles in natural flavoring, pharmaceutical production, and the food industry [1,2]. According to data from the Food and Agriculture Organization (FAO), the global production of lemons and limes reached 23.201 million tons in 2024. Notably, the production proportions of Eureka, Perfume, and Sweet lemon varieties have exhibited a steady upward trend in recent years. Traditionally, the economic sustainability of the lemon industry relies heavily on the direct sale of fresh fruits. However, there is an increasingly urgent need to extend the industrial chain through deep processing to enhance its economic value. At present, lemon-derived products, including fruit juice, pectin, and essential oils, have already achieved industrial-scale production. Nevertheless, the sustainable development of the lemon industry is severely constrained by prominent issues such as excessive product homogenization and low added value. Against this backdrop, lemon-fermented fruit wine, which is recognized for its health-promoting properties and cultural connotations, has emerged as a promising and rapidly developing segment in the global fruit wine market. In particular, it is highly favored by young consumers due to its low alcohol content, refreshing flavor, and inherent natural antioxidant properties, thereby providing a new pathway for the high-value utilization of lemons.

Fruit wine is broadly defined as an alcoholic beverage obtained by the alcoholic fermentation of non-grape fruit juices [3]. Fermentation is a pivotal process in fruit wine production, as it directly determines the flavor quality of the final product. During lemon juice fermentation, diverse flavor compounds, with volatile compounds being the most important, are generated through the metabolic activity of *Saccharomyces cerevisiae* [4]. Notably, fruit cultivar is a key factor that significantly influences the aroma profile and volatile compound quality of fermented fruit wine [5]—a phenomenon that is particularly prominent in lemon-fermented fruit wine, given the distinct physicochemical differences among major lemon varieties. In China, three major commercial lemon varieties exhibit distinctive physicochemical properties, which directly regulate the metabolic pathways of yeast and the formation of flavor compounds during fermentation: Sweet lemon is characterized by low acidity and high sugar content; Perfume lemon is abundant in volatile compounds; and the conventional Eureka lemon possesses high acidity and a pronounced fruity aroma. Previous studies [6] have demonstrated that the elevated sugar content in Sweet Lemon can promote ester biosynthesis, while its low acidity may inhibit the release of certain aroma precursors. Although the complex terpenoid compounds in Lemon contribute to its distinctive floral aroma, the oxidative sensitivity of these terpenoids may compromise the flavor stability of the fermented wine [6,7].

The fruit cultivar significantly influences the aroma profile and quality of volatile compounds in fermented fruit wine. However, there is a lack of systematic and comprehensive analysis of the flavor profiles and volatile compound compositions associated with different lemon types in lemon-fermented fruit wine. This research gap not only limits the in-depth understanding of the correlation between lemon cultivar characteristics and fermentation-derived flavor formation but also restricts the development of targeted products to meet consumer demand for diversified flavor experiences—thus highlighting the necessity of the present study focusing on flavor differences among fermented wines from different lemon varieties. In this study, lemon-fermented wine produced from Eureka, Sweet, and Perfume lemons were selected for investigation. Flavor components were qualitatively and quantitatively analyzed using solid-phase microextraction (SPME) combined with gas chromatography–mass spectrometry (GC–MS) and multidimensional GC–MS (MDGC–MS). Odor activity values (OAVs) were employed to identify the characteristic flavor compounds in the three lemon cultivar-based fermented fruit wines, and multivariate statistical approaches including orthogonal partial least squares discriminant analysis (OPLS-DA) was applied to examine the relationship between fruit wine flavor quality and lemon cultivar. The objective was to elucidate the flavor formation mechanisms of different lemon-fermented fruit wine, thereby providing a theoretical foundation for raw material selection, process optimization, and differentiated product development.

## 2. Materials and Methods

### 2.1. Samples and Lemon-Fermented Fruit Wine Preparation

Three lemon varieties (Eureka, Sweet, and Perfume lemons) cultivated in China were selected as raw materials, which were all harvested from orchards in Tongnan, Chongqing, in June 2024. The basic chemical parameters of the different types of lemons are shown in Table S1. The lemon samples were stored at 4 °C prior to processing. The lemons were then washed, dried, cut, and juiced using a mechanical juicer. Fermentation was conducted using the yeast strain CEC01 according to the method described by Lu et al. [8]. Select lemons of uniform size, add three times their volume of pure water to extract juice. Use a gauze to filter out the residue. Adjust the sugar content to 20% (mass fraction, the same as below) using white granulated sugar. Heat at 70 °C for 5 min for moderate pasteurization pretreatment, then cool to below 30 °C. Add 0.12% activated yeast (CEC01) on a mass fraction basis (*w*/*w*) and incubate in a 32 °C incubator for 10 days. Fermentation was regarded as complete when the °Brix remained stable for two consecutive measurements, and the total fermentation duration was 10 days. After the fermentation is complete, filter and take the supernatant for measurement. The supernatants were sealed immediately and preserved at −20 °C for subsequent analysis. Each cultivar underwent three repetitions of fermentation. Each parallel batch fermentation is carried out with a volume of 500 mL. Eureka lemon-fermented wine (ELFW), Sweet lemon-fermented wine (SLFW), and Perfume lemon-fermented wine (PLFW) were kept at −20 °C until analytical analysis. The alcohol contents of ELFW, SLFW, and PLFW were 0.4 ± 0.1%, 1.7 ± 0.2%, and 3.1 ± 0.1% (*v*/*v*), respectively (Table S2). Fermentation experiments were performed with one batch of lemon raw material, and three parallel fermentations were set as technical replicates.

### 2.2. Reagents and Chemicals

The yeast strain CEC01 was obtained from Hubei Anqi Yeast Co. (Yichang, China). Granulated sugar was purchased from a local supermarket. Glucose (99.7%) was supplied by Shandong Xiya Chemical Reagent Co. (Linyi, China). A standard mixture of C5–C20 n-alkanes was obtained from Sigma-Aldrich Co. (Shanghai, China), and cyclohexanone (99%) was sourced from Shanghai Aladdin Biochemical Technology Co. (Shanghai, China).

### 2.3. Solid-Phase Microextraction (SPME)

Volatile compounds were extracted following the method described by Cheng et al. [9]. A 5 mL aliquot of lemon-fermented fruit wine was transferred into a 20 mL vial, followed by the addition of a magnetic stir bar and the internal standard cyclohexanone. The mixture was thoroughly shaken and sealed using a polytetrafluoroethylene-lined cap. The vial was equilibrated in a 40 °C water bath for 20 min. Volatile compounds were subsequently extracted using a SPME fiber with 50/30 μm divinylbenzene/carbon molecular sieve/polydimethylsiloxane (DVB/CAR/PDMS) (Supelco, Bellefonte, PA, USA) for 30 min. Each sample was performed in triplicate.

### 2.4. Gas Chromatography–Mass Spectrometry (GC–MS) Analysis

Volatile compounds in three lemon cultivar-based fermented fruit wines were analyzed using an Agilent 7890B GC-5977A MS system (Agilent, Palo Alto, CA, USA). Helium was used as the carrier gas at a flow rate of 1.2 mL/min. Splitless injection was performed with the GC inlet temperature set to 230 °C. The extracted SPME fibers were inserted into the GC inlet for 5 min. Volatile compound separation was achieved using a DB-5 column (30 m × 0.25 mm i.d. × 0.25 μm) (Agilent, Palo Alto, CA, USA). The column oven temperature was initially held at 35 °C for 3 min, then increased to 203 °C at a rate of 6 °C/min, and further increased to 243 °C at 10 °C/min. The EI ion source was operated at 70 eV with a transfer line temperature of 280 °C, and the mass spectra were acquired over an *m*/*z* range of 33–500 amu.

### 2.5. Multidimensional GC–MS (MDGC–MS) Analysis

Volatile compounds in the three lemon cultivar-based fermented fruit wines were analyzed using an Agilent 7890B MDGC-5977B MS system (Agilent, Palo Alto, CA, USA). The method was carried out as described previously [10]. A non-polar BP-5 column (^1^D, 30 m × 0.53 mm i.d. × 0.5 μm) (SGE, Pflugerville, TX, USA) and a polar SOLgel-wax column (^2^D, 30 m × 0.53 mm i.d. × 0.5 μm) (SGE, Pflugerville, TX, USA) were employed for compound separation. The carrier gas flow rates for ^1^D and ^2^D were 8.7345 and 8.7663 mL/min, respectively. The sample extraction procedure, carrier gas, transfer line temperature, ion source temperature, and *m*/*z* scanning range were consistent with those used in the GC–MS analysis.

### 2.6. Qualitative and Semi-Quantitative Detection

Qualitative analysis of volatile compounds in the lemon-fermented fruit wine was performed by comparing the calculated linear retention index (LRI) values with the literature-reported LRI data and by matching total ion chromatograms with those in the NIST11 and W10N14 mass spectral libraries. LRI qualitative system is originally established for single-column GC–MS. A semi-quantitative analysis was conducted using cyclohexanone as the internal standard.

### 2.7. Odor Activity Values (OAVs)

OAVs were calculated as the ratio of the concentrations of volatile compounds in lemon-fermented fruit wine to their corresponding odor thresholds, which are usually reported in the relevant literature. Compounds with OAVs ≥ 1 were identified as characteristic flavor compounds that contributed significantly to the overall aroma profile.

### 2.8. Statistical Analysis

Qualitative analysis of volatile compounds was performed using the MSD ChemStation software (Version F.01.01.2317). Data visualization was performed using OriginPro 2021. OPLS-DA was performed using SIMCA 14.1. The figures were plotted using Origin 7.5 (OriginLab, Northampton, MA, USA).

## 3. Results and Analysis

### 3.1. Qualitative Analysis of Volatile Compounds in Three Lemon Cultivar-Based Fermented Fruit Wines

Volatile compounds in ELFW, SLFW, and PLFW were extracted and concentrated using SPME and subsequently analyzed using GC–MS and MDGC–MS. As shown in Table 1, 85 volatile compounds were identified across three lemon cultivar-based fermented fruit wines, comprising 31 alcohols, 25 hydrocarbons, 18 esters, three ketones, three acids, two aldehydes, and three other compounds. The quantity and diversity of volatile compounds identified herein differ from those reported by Wen et al. [11], mainly due to inconsistent yeast strains and fermentation protocols applied in the two studies. Distinct microbial metabolic characteristics and processing conditions jointly lead to the divergent volatile profiles between the two lemon wine systems. In this study, more volatile compounds were detected, which may be attributed to the enhanced separation of monoterpenoid isomers and the improved sensitivity, accuracy, and repeatability offered by MDGC–MS [12]. The integration of multiple analytical platforms enhanced the comprehensiveness of volatile compounds detection in lemon wine. Cheng et al. [13] demonstrated improved efficiency of volatile compound identification in citrus juice by combining GC–MS/PFPD, MDGC–MS/O, and GC–FID/O. However, the combined application of GC–MS and MDGC–MS remains rare in fermented wine analysis. In this study, 66 volatile compounds were detected by GC–MS and 59 by MDGC–MS, with 40 compounds identified using both techniques. Additionally, 26 volatile compounds were exclusively detected by GC–MS and 19 solely by MDGC–MS, and all aldehydes and acids were uniquely detected by MDGC–MS.

**Figure 1:**
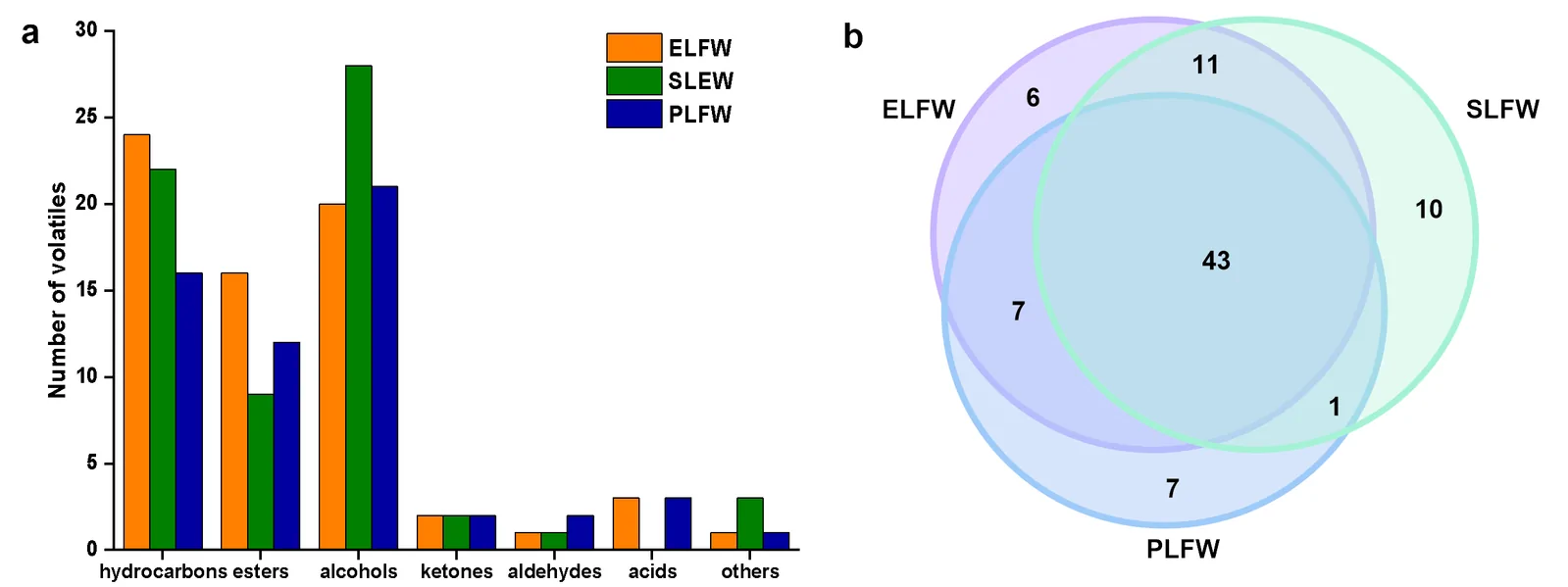
Comparison (**a**) and Venn diagram (**b**) of volatile compounds in the three lemon cultivar-based fermented fruit wines.

The variations in the brewing raw materials also resulted in differences in the volatile compound profiles among the lemon-fermented fruit wine. A total of 67, 65, and 58 volatile compounds were identified in ELFW, SLFW, and PLFW, respectively. As shown in Figure 1a, ELFW contained the highest number of hydrocarbons (24) and esters (16). SLFW exhibited the highest abundance of alcohols (28) and hydrocarbons (22), whereas PLFW had the highest levels of alcohols (22) and hydrocarbons (16). Figure 1b shows the Venn diagram illustrating the distribution of volatile compounds identified in ELFW, SLFW, and PLFW. A total of 43 volatile compounds were shared by all three lemon-fermented wines, representing the core aroma components of lemon-fermented wines. Notably, the enantiomeric assignment of (-)-β-pinene in this study is an inference integrated from mass spectrum matching and published matrix background data, which has not been verified by chiral column chromatography. For cultivar-exclusive volatiles, six unique compounds were detected only in ELFW, 10 exclusive volatiles were specific to SLFW, and seven unique components were found solely in PLFW. Three acids were detected in both ELFW and PLFW, but none were observed in SLFW. Differences in lemon cultivars are a major factor contributing to the distinct volatile profiles of fermented wines, while multiple fermentation-related factors jointly modulate the final aroma composition. Additionally, six volatile compounds, including eremophilene, isodurene, ethyl nonanoate, nonyl acetate, ethyl isobutyrate, and ethyl dodecanoate, were unique to the ELFW. The inherent sesquiterpenes of Eureka lemon were retained during the fermentation process, while the medium- and long-chain fatty acid esters are mainly produced by yeast-mediated esterification of fatty acids and alcohols during fermentation [14,15]. SLFW exhibited a notably high abundance of C6 green leaf alcohols and oxygenated monoterpenes. Furthermore, a distinct set of ten volatile compounds, such as 3-hexen-1-ol, 1-hexanol, γ-isogeraniol, cis-isogeraniol, (E)-3-hexen-1-ol, (Z)-3-hexen-1-ol, cis-p-2-menthen-1-ol, methyl benzoate, carvacrol, and 1,8-cineole, were detected only in the SLFW. Seven volatile compounds, including cis-α-bisabolene, trans-carveol, citronellol, isopregol, ethyl octanoate, d-carvone, and octanal, were specific to PLFW, which also accumulated typical citrus monoterpenoids and aromatic aldehydes. Obvious qualitative compositional variations of volatile profiles were observed among three lemon cultivar-based fermented fruit wines, which was consistent with the findings in other fruit wine, such as grape [16], apricot [5], jujube [17], and sugarcane [18]. These results confirmed that fruit cultivar played a critical role in shaping wine flavor profiles.

### 3.2. Semi-Quantitative Analysis of Volatile Compounds in Three Lemon Cultivar-Based Fermented Fruit Wines

**Figure 2:**
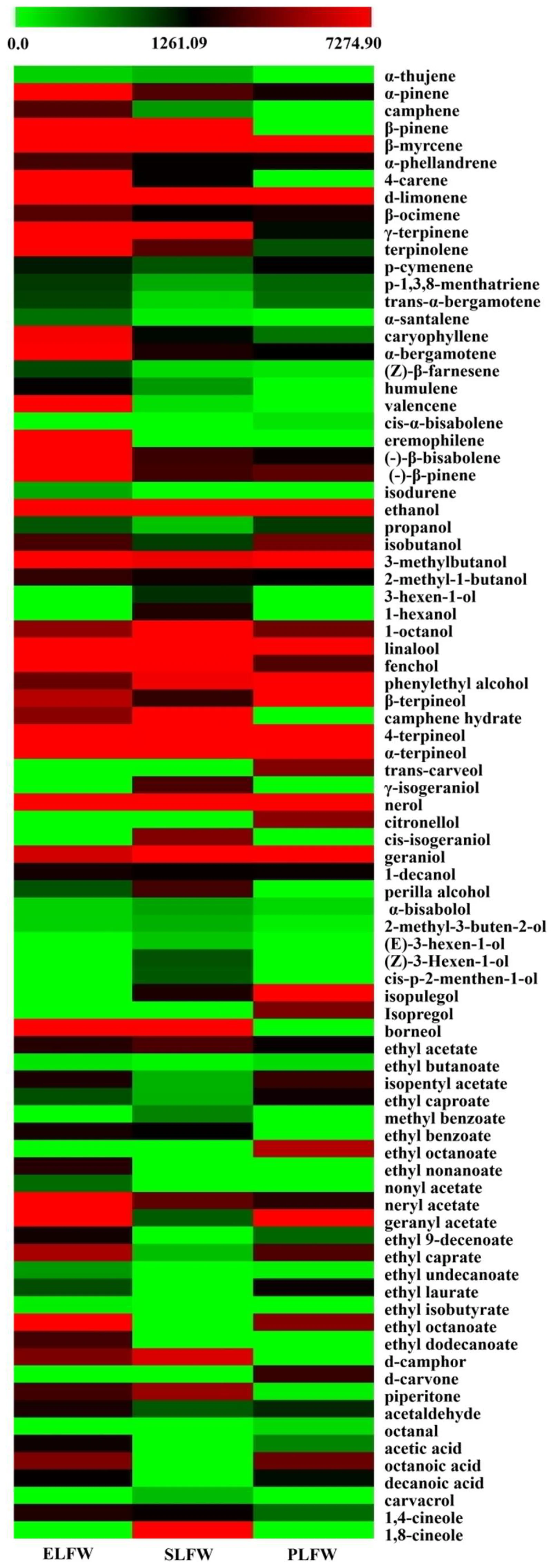
Heat-map illustrating the changes of volatile compounds in the three lemon cultivar-based fermented fruit wines.

The flavor profile of fruit wine is directly determined by its composition of volatile compounds. As illustrated in Figure 2 and Table S3, the ELFW contained the highest total content of volatile compounds, which was 1.35-fold and 1.92-fold higher than those of SLFW and PLFW, respectively. Such pronounced differences could be primarily attributed to varietal variations among the raw materials. The three fermented fruit wines derived from different lemon cultivars exhibited similar distribution profiles of volatile compounds. Alcohols constituted the predominant chemical class across all samples, accounting for 51.25%, 73.50%, and 57.28% of the total volatiles in ELFW, SLFW, and PLFW, respectively. This observation aligns with the central role of alcohols as major metabolic products generated during yeast-mediated alcoholic fermentation [19]. Subsequent classes in terms of relative abundance included hydrocarbons (22.15–40.85%) and esters (1.40–6.10%). In contrast, aldehydes (0.11–0.24%), ketones (0.50–1.52%), organic acids (0.00–1.07%), and other miscellaneous volatiles (0.14–1.31%) were present at comparatively low concentrations. The relatively low concentrations of aldehydes, ketones, and organic acids can be attributed to two main plausible potential mechanisms: firstly, these compounds often serve as intermediate metabolites during fermentation and are readily further metabolized by yeast (e.g., aldehydes are reduced to alcohols, organic acids participate in esterification reactions) [20]; secondly, due to their high volatility, they tend to be carried away by CO_2_ during the fermentation process, which could contribute to their reduced final concentrations. In ELFW, significantly higher concentrations of α-terpineol (210,225.72 ± 6846.23 μg/L), d-limonene (180,524.74 ± 753.88 μg/L), 4-terpineol (115,855.27 ± 12,369.72 μg/L), γ-terpinene (66,317.34 ± 767.85 μg/L), and fenchol (44,654.16 ± 3310.50 μg/L) were detected. By contrast, the most abundant volatile compounds in SLFW were 4-terpineol (159,796.47 ± 7102.88 μg/L), α-terpineol (137,578.70 ± 4013.42 μg/L), d-limonene (93,249.45 ± 15,300.22 μg/L), nerol (64,153.28 ± 1660.29 μg/L), and linalool (47,260.68 ± 1546.87 μg/L). For PLFW, the dominant volatile components were d-limonene (150,878.36 ± 20,803.96 μg/L), α-terpineol (90,787.51 ± 1685.06 μg/L), linalool (40,440.15 ± 2084.07 μg/L), 3-methylbutanol (28,915.73 ± 1804.97 μg/L), and 4-terpineol (25,704.50 ± 620.23 μg/L). Notably, d-limonene, as a characteristic volatile compound of citrus fruits [21,22], is a dominant component in all three fruit wines. However, its content varies significantly among them (ELFW > PLFW > SLFW), further confirming that different lemon varieties accumulate varying amounts of d-limonene. Although this compound undergoes slight transformation during the fermentation process, it largely retains the varietal specificity of the raw material. Terpene alcohols such as α-terpineol and 4-terpineol are relatively stable during the fermentation process, and their content depends primarily on the initial content in the raw materials. Moreover, the relatively high content of 3-methylbutanol in PLFW may be related to the higher leucine content (the precursor amino acid of 3-methylbutanol) in the raw material of this lemon cultivar. Yeast converts leucine into 3-methylbutanol through the Ehrlich pathway [23], and differences in the precursor substances in the raw material directly lead to differences in the final product content.

### 3.3. Analysis of OAVs of Key Volatile Compounds in Three Lemon Cultivar-Based Fermented Fruit Wines

The odor activity values (OAVs), defined as the ratio of the concentration of individual volatile compounds to their respective sensory thresholds, was calculated for the three fermented fruit wines derived from different lemon cultivars. Volatile compounds with an OAV ≥ 1 were preliminarily regarded as aroma-active compounds to the aroma of fruit wines, since this threshold value indicates that the compounds are present at concentrations sufficient to be perceived by the human olfactory system. Based on this criterion, the characteristic flavor compounds and differences in aroma profiles among the three lemon fermented fruit wines were preliminarily screened for reference. As illustrated in Table 2, a total of 46 volatile compounds with OAV ≥ 1 were identified, which were classified into seven chemical categories: 14 esters, 13 hydrocarbons, 12 alcohols, three ketones, two organic acids, one aldehyde, and two other compounds. Among these potential characteristic flavor candidates, esters accounted for the most prominent category, which is consistent with the findings reported by Zhao et al. [24] in their research on fruit wines fermented with different kiwifruit varieties, thereby further confirming the crucial role of esters in constructing the aroma profile of fermented fruit beverages. The number of characteristic compounds (with OAV ≥ 1) differed significantly among the three fruit wine varieties, with 38, 33, and 33 such compounds identified in ELFW, SLFW, and PLFW samples, respectively. Specifically, in the ELFW, α-pinene, β-myrcene, d-limonene, linalool, fenchol, α-terpineol, neryl acetate, and 1,4-cineole were identified as the dominant characteristic compounds (OAVs > 400). Similarly, d-limonene, linalool, fenchol, α-terpineol, geraniol, and 1,4-cineole were the major characteristic compounds in the SLFW, exhibiting high abundance comparable to the dominant compounds in ELFW. In contrast, the key characteristic compounds contributing to the flavor of PLFW were fenchol, linalool, d-limonene, ethyl octanoate, and 1,4-cineole, which differed slightly from those of the other two varieties. As a monoterpene alcohol, fenchol is primarily formed through the metabolic transformation of terpenoid precursors catalyzed by yeast during the fermentation process of fruit wine. Wu et al. [6] reported that fenchol production varied depending on the yeast strain, with 71B producing the highest levels, followed by NM-8 and DV10. The monoterpenoids of α-terpineol, linalool, and d-limonene are primarily derived from citrus raw materials and contribute to the floral and fruity aromas of lemon wine [13]. Known for its fruity note, ethyl octanoate is another important compound. According to OAVs, 23 characteristic flavor compounds were identified across lemon-fermented fruit wine, with isoamyl acetate (banana aroma) and isoamyl alcohol (alcoholic aroma) ranked among the most influential [25].

### 3.4. Multivariate Statistical Analysis of Three Lemon Cultivar-Based Fermented Fruit Wines

**Figure 3:**
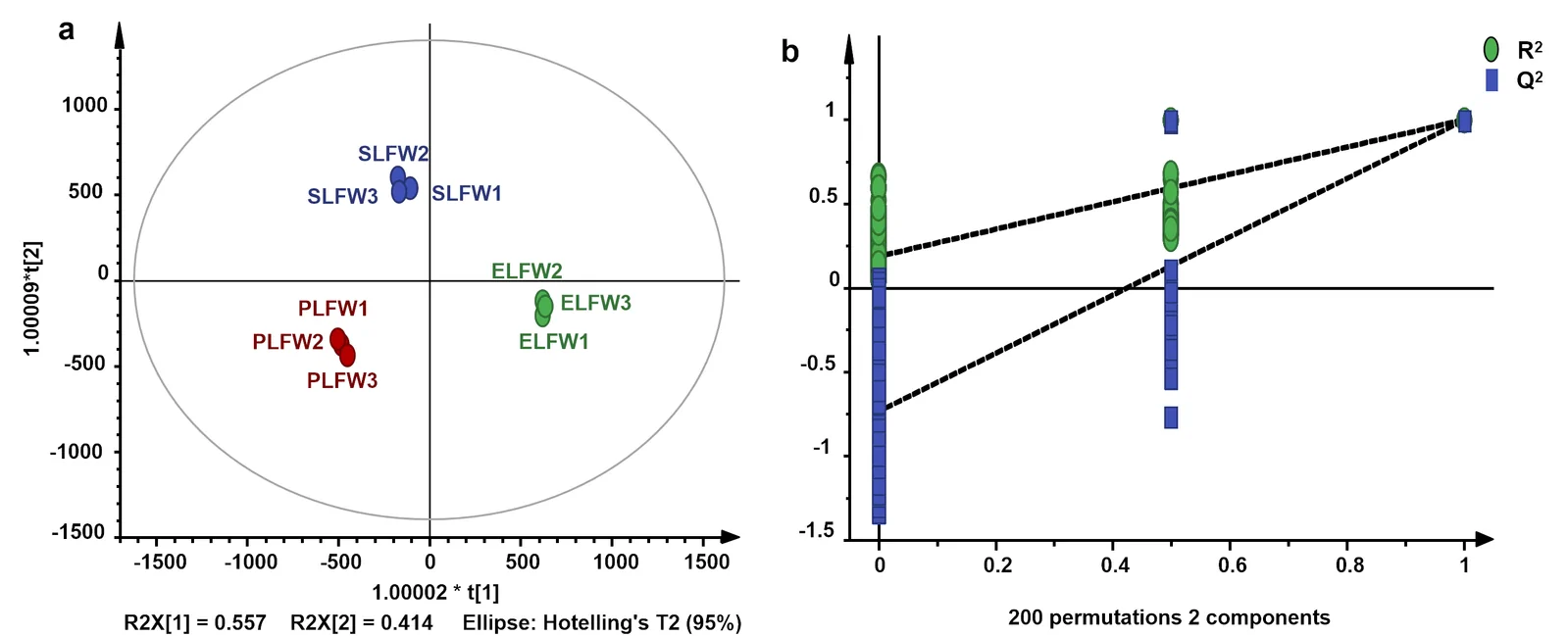

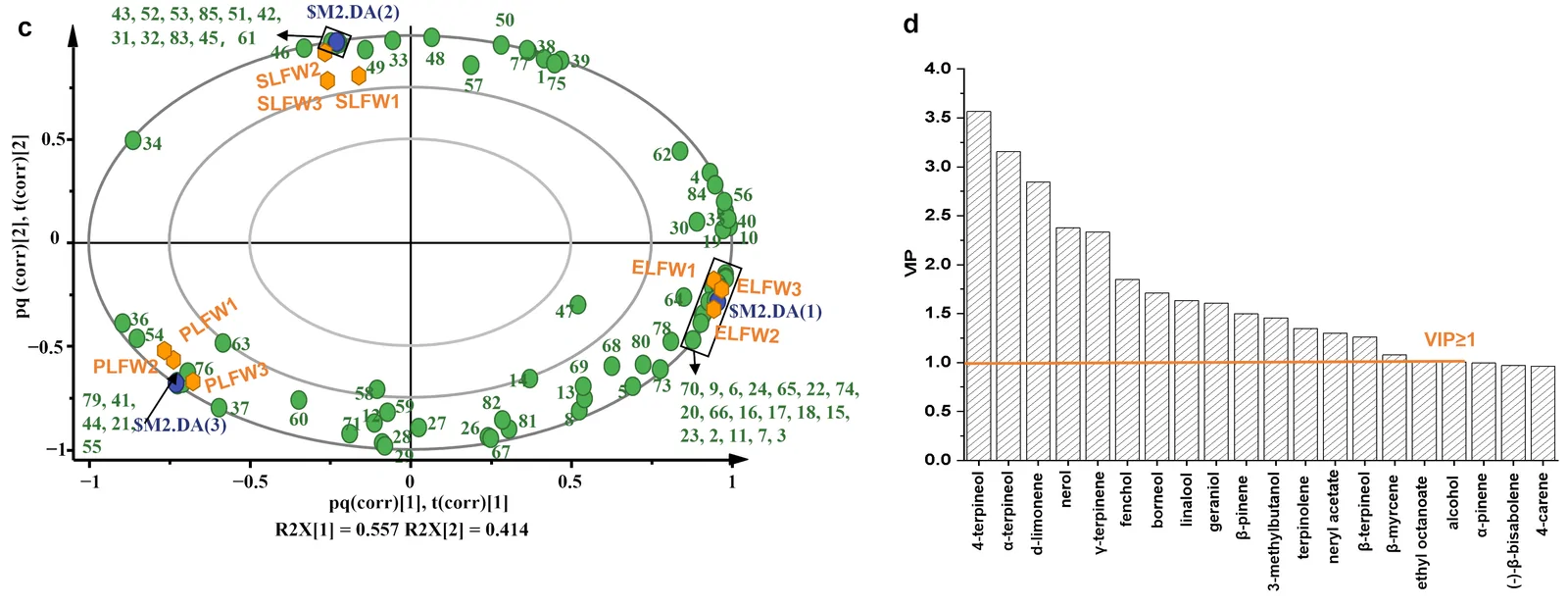
Multivariate statistical analysis of volatile compounds in three lemon cultivar-based fermented fruit wines ((**a**): OPLS-DA score, (**b**): permutation test, (**c**): OPLS-DA biplot, (**d**): VIP), the numerical suffixes 1, 2, and 3 of SLFW, ELFW, and PLFW represent three independent biological fermentation replicates for each cultivar.

In this study, volatile compounds in three lemon cultivar-based fermented fruit wines were analyzed using OPLS-DA, which is a supervised multivariate statistical technique capable of identifying inter-sample differences and extracting characteristic markers [16]. Data analysis was based on a matrix of the content of volatile compounds (X variables) and lemon-fermented fruit wine types (Y variable). Figure 3a illustrates that the cumulative variance of R2X reached 97.1%, indicating that the model effectively captured the primary characteristics of the samples. A clear separation was observed among the three groups of lemon cultivar-based fermented fruit wines, which not only reflected the distinct varietal differences in volatile compound compositions but also enabled satisfactory classification of the fruit wines according to their lemon raw material types. To assess model reliability and exclude overfitting, 200 permutation tests were conducted. The R^2^ and Q^2^ intercepts were 0.184 and −0.751, respectively, confirming the model’s robustness (Figure 3b). The OPLS-DA biplot (Figure 3c) revealed associations between volatile compounds and lemon varieties. The ELFW was primarily correlated with ethyl nonanoate, ethyl undecanoate, (Z)-β-ocimene, α-phellandrene, (-)-β-pinene, nonyl acetate, eremophilene, ethyl dodecanoate, valencene, caryophyllene, neryl acetate, α-bergamotene, (Z)-β-farnesene, α-santalene, (-)-β-bisabolene, α-pinene, terpinolene, 4-carene, and camphene. The SLFW was associated with nerol, (Z)-3-hexen-1-ol, cis-p-2-menthen-1-ol, γ-isogeraniol, (E)-3-hexen-1-ol, 1-hexanol, carvacrol, cis-isogeraniol, methyl benzoate, geraniol, 1-octanol, and α-bisabolol. PLFW was primarily associated with phenylethyl alcohol, isopulegol, trans-carveol, citronellol, cis-α-bisabolene, and isopregol.

The variable importance in projection (VIP) values was employed to quantify the contribution of individual volatile compounds to the classification performance of the OPLS-DA model. A higher VIP value indicates a stronger influence of the corresponding volatile compound on the inter-group discrimination of the model. Specifically, volatile compounds with VIP > 1 were regarded as significant contributors to the overall flavor profile of the fruit wines [46,47], and these compounds were also considered as potential characteristic volatile markers [48]. As shown in Figure 3d, a total of 17 flavor markers were identified as the key variables responsible for distinguishing the three lemon cultivar-based fermented fruit wines. These key characteristic volatile compounds included 4-terpineol, α-terpineol, d-limonene, nerol, γ-terpinene, fenchol, borneol, linalool, geraniol, β-pinene, 3-methylbutanol, terpinolene, neryl acetate, β-terpineol, β-myrcene, ethyl octanoate, and ethanol.

These 17 differential markers are potential contributors to inter-sample aroma divergence and can assist in distinguishing flavor characteristics of different lemon-fermented wines. Their concentration differences reflect the varietal traits of raw lemon materials (e.g., limonene abundance) as well as the metabolic capacity of yeast during fermentation (including the synthesis of monoterpene alcohols and esters). Accordingly, the concentration discrepancies of these compounds provide a critical chemical reference for differentiating the three lemon-fermented wine samples.

## 4. Conclusions

SPME combined with GC–MS and MDGC–MS was employed to analyze volatile compounds in ELFW, SLFW, and PLFW. A total of 85 volatile compounds were identified, with 66 detected by GC–MS and 59 by MDGC–MS. The integration of these two complementary analytical techniques effectively enhanced the comprehensiveness and accuracy of volatile compound profiling, thereby ensuring the thorough characterization of volatile components in the three lemon cultivar-based fermented fruit wines. Specifically, 67, 65, and 58 volatile compounds were identified in ELFW, SLFW, and PLFW, respectively. Moreover, six, 10, and seven volatile compounds were detected uniquely in the ELFW, SLFW, and PLFW, respectively. These results clearly demonstrated the distinct differences in the composition and quantity of volatile compounds among the three lemon fruit wines, which are closely associated with their inherent varietal characteristics. Alcohols constituted the predominant volatile compounds group (51.25–73.50%), followed by hydrocarbons (22.15–40.85%) and esters (1.40–6.10%). The volatile compound composition varied among lemon wine types. Moreover, α-terpineol, 4-terpineol, and d-limonene were the dominant compounds in the ELFW, SLFW, and PLFW, respectively. Substantial differences were observed in the number, type, and concentration of volatile compounds among the three lemon cultivar-based fermented fruit wines. A total of 46 characteristic flavor compounds in fruit wine were identified based on OAV analysis. Among them, fenchol, d-limonene, and 1,4-cineole were the most prominent in ELFW, while fenchol, linalool, and d-limonene were the dominant compounds in both SLFW and PLFW. Additionally, 17 flavor markers were identified through multivariate statistical analysis using OPLS-DA combined with VIP values, enabling clear differentiation among the three lemon cultivar-based fermented fruit wines. Lemon fruit wine samples were prepared via microbrewing. By combining SPME with GC–MS and MDGC–MS, the shared and differential volatile compounds profiles of the three lemon cultivar-based fermented fruit wines were comprehensively revealed. Multivariate data analysis enabled the identification of key flavor markers that distinguished the three wine types. Moreover, the divergent matrix physicochemical properties (final TSS, ethanol, residual glucose and citric acid) among three lemon wines serve as critical background factors modulating volatile profiles and OAV performance. Ethanol concentration alters the solubility and partitioning behavior of aroma compounds; organic acids and residual sugars influence yeast secondary metabolic pathways for ester, terpene, and alcohol synthesis. The OPLS-DA multivariate separation of three fermented samples is partially driven by these cultivar-specific physicochemical differences, which jointly explain the overall variations in volatile composition and odor activity values. Although these identifiers were successfully characterized, the specific contributions of individual flavor compounds, their concentrations, interactions, and regulatory mechanisms may require further investigation. Such studies are essential to support the theoretical foundation and technical pathways for precise flavor design of lemon-fermented fruit wine. This study provides a scientific basis for flavor identification and regulation strategies and offers a valuable reference for the development and promotion of differentiated lemon-fermented fruit wine products.

## Figures and Tables

**Table 1:** Identification analysis of volatiles in the three lemon cultivar-based fermented fruit wines.

No.	Compounds	LRI- Calculated	LRI-Referenced ^a^	Identification		Samples		
				GC-MS	MDGC-MS	ELFW	SLFW	PLFW
	**Hydrocarbons**							
1	α-thujene	936	928	MS, LRI	MS	■	■	-
2	α-pinene	945	934	MS, LRI	MS	■	■	■
3	camphene	961	950	MS, LRI	MS	■	■	-
4	β-pinene	991	978	MS, LRI	MS	■	■	-
5	β-myrcene	1000	990	MS, LRI	-	■	■	■
6	α-phellandrene	1017	1005	MS, LRI	MS	■	■	■
7	4-carene	1027	1014	MS, LRI	MS	■	■	-
8	d-limonene	1043	1030	MS, LRI	MS	■	■	■
9	(Z)-β-ocimene	1060	1046	MS, LRI	MS	■	■	■
10	γ-terpinene	1075	1059	MS, LRI	MS	■	■	■
11	terpinolene	1098	1087	MS, LRI	MS	■	■	■
12	p-cymenene	1100	1089	MS, LRI	MS	■	■	■
13	p-1,3,8-menthatriene	1121	1112	MS, LRI	MS	■	■	■
14	trans-α-bergamotene	1424	1432	MS, LRI	-	■	■	■
15	α-santalene	1431	1420	MS, LRI	-	■	■	-
16	caryophyllene	1436	1426	MS, LRI	-	■	■	■
17	α-bergamotene	1445	1434	MS, LRI	MS	■	■	■
18	(Z)-β-farnesene	1459	1444	MS, LRI	MS	■	■	■
19	humulene	1471	1451	MS, LRI	MS	■	■	-
20	valencene	1508	1494	MS, LRI	MS	■	■	-
21	cis-α-bisabolene	1509	1493	MS, LRI	-	-	-	■
22	eremophilene	1509	1490	MS, LRI	-	■	-	-
23	(-)-β-bisabolene	1520	1525	MS, LRI	MS	■	■	■
24	(-)-β-pinene	-	-	-	MS	■	■	■
25	isodurene	-	-	-	MS	■	-	-
	**Alcohols**							
26	ethanol	567	551	MS, LRI	MS	■	■	■
27	propanol	593	595	MS, LRI	-	■	■	■
28	isobutanol	635	650	MS, LRI	MS	■	■	■
29	3-methylbutanol	746	740	MS, LRI	MS	■	■	■
30	2-methyl-1-butanol	748	741	MS, LRI	-	■	■	■
31	3-hexen-1-ol	864	856	MS, LRI	-	-	■	-
32	1-hexanol	879	870	MS, LRI	MS	-	■	-
33	1-octanol	1083	1073	MS, LRI	MS	■	■	■
34	linalool	1110	1100	MS, LRI	MS	■	■	■
35	fenchol	1134	1119	MS, LRI	MS	■	■	■
36	phenylethyl alcohol	1142	1126	MS, LRI	MS	■	■	■
37	β-terpineol	1162	1144	MS, LRI	MS	■	■	■
38	camphene hydrate	1166	4450	MS, LRI	-	■	■	-
39	4-terpineol	1196	1178	MS, LRI	MS	■	■	■
40	α-terpineol	1215	1191	MS, LRI	MS	■	■	■
41	trans-carveol	1228	1217	MS, LRI	-	-	-	■
42	γ-isogeraniol	1230	1222	MS, LRI	-	-	■	-
43	nerol	1240	1228	MS, LRI	MS	■	■	■
44	citronellol	1244	1228	MS, LRI	MS	-	-	■
45	cis-isogeraniol	1252	1254	MS, LRI	-	-	■	-
46	geraniol	1259	1256	MS, LRI	MS	■	■	■
47	1-decanol	1278	1270	MS, LRI	-	■	■	■
48	perilla alcohol	1310	1299	MS, LRI	-	■	■	■
49	α-bisabolol	1697	1682	MS, LRI	-	■	■	■
50	2-methyl-3-buten-2-ol	-	-	-	MS	■	■	■
51	(E)-3-hexen-1-ol	-	-	-	MS	-	■	-
52	(Z)-3-Hexen-1-ol	-	-	-	MS	-	■	-
53	cis-p-2-menthen-1-ol	-	-	-	MS	-	■	-
54	isopulegol	-	-	-	MS	-	■	■
55	isopregol	-	-	-	MS	-	-	■
56	borneol	-	-	-	MS	■	■	-
	**Esters**							
57	ethyl acetate	621	618	MS, LRI	MS	■	■	■
58	ethyl butanoate	806	801	MS, LRI	MS	■	■	■
59	isopentyl acetate	886	879	MS, LRI	MS	■	■	■
60	ethyl caproate	1008	999	MS, LRI	MS	■	■	■
61	methyl benzoate	1102	1094	MS, LRI	-	-	■	-
62	ethyl benzoate	1178	1171	MS, LRI	-	■	■	-
63	ethyl octanoate	1200	1191	MS, LRI	MS	-	-	■
64	ethyl nonanoate	1299	1294	MS, LRI	MS	■	-	-
65	nonyl acetate	1313	1310	MS, LRI	-	■	-	-
66	neryl acetate	1362	1364	MS, LRI	MS	■	■	■
67	geranyl acetate	1381	1383	MS, LRI	MS	■	■	■
68	ethyl 9-decenoate	1391	1389	MS, LRI	-	■	-	■
69	ethyl caprate	1399	1392	MS, LRI	MS	■	■	■
70	ethyl undecanoate	1498	1496	MS, LRI	-	■	-	■
71	ethyl laurate	1599	1592	MS, LRI	-	■	-	■
72	ethyl isobutyrate	-	-	-	MS	■	-	-
73	methyl octanoate	-	-	-	MS	■	-	-
74	ethyl dodecanoate	-	-	-	MS	■	-	-
	**Ketones**							
75	d-camphor	1158	1143	MS, LRI	MS	■	■	-
76	d-carvone	1253	1256	MS, LRI	-	-	-	■
77	piperitone	1270	1256	MS, LRI	-	■	■	■
	**Aldehydes**							
78	acetaldehyde	-	-	-	MS	■	■	■
79	octanal	-	-	-	MS	-	-	■
	**Acids**							
80	acetic acid	-	-	-	MS	■	-	■
81	octanoic acid	-	-	-	MS	■	-	■
82	decanoic acid	-	-	-	MS	■	-	■
	**Others**							
83	carvacrol	1306	1298	MS, LRI	-	-	■	-
84	1,4-cineole	-	-	-	MS	■	■	■
85	1,8-cineole	-	-	-	MS	-	■	-

^a^: LRIs were taken from the flavor database: https://www.vcf-online.nl/VcfCompoundSearch.cfm (accessed on 17 August 2026), http://www.odour.org.uk/index.html (accessed on 17 August 2026). ■ Volatile compounds have been identified in this cultivar. “-” indicates that no corresponding LRI values were found or the compound was not detected in the cultivar.

**Table 2:** OAVs of volatiles in the three OAVs of volatiles in the three lemon cultivar-based fermented fruit wines.

Compounds	Threshold (μg/L)	Reference	OAVs		
			ELFW	SLFW	PLFW
α-pinene	9.5 ^w^	[26]	1339.49	333.88	186.40
β-pinene	140 ^w^	[27]	189.83	115.10	-
β-myrcene	36 ^w^	[26]	575.71	214.54	383.67
α-phellandrene	40 ^w^	[27]	71.13	35.52	39.33
d-limonene	60 ^w^	[26]	3008.75	1554.16	2514.64
(Z)-β-ocimene	34 ^w^	[28]	96.68	40.01	51.89
γ-terpinene	1000 ^w^	[27]	66.32	25.40	1.20
terpinolene	200 ^w^	[27]	104.93	16.69	4.26
p-cymenene	85 ^w^	[29]	13.46	9.91	15.60
p-1,3,8-menthatriene	15 ^w^	[29]	65.00	27.90	51.03
caryophyllene	64 ^w^	[27]	111.46	19.21	10.75
humulene	160 ^w^	[27]	8.83	3.18	-
(-)-β-pinene	4160 ^w^	[30]	3.02	<1	<1
3-methylbutanol	6500 ^e^	[31]	3.39	1.07	4.45
linalool	15 ^e^	[32]	1163.48	3150.71	2696.01
fenchol	2.1 ^a^	[33]	21,263.88	10,036.28	1530.33
4-terpineol	5000 ^e^	[34]	23.17	31.96	5.14
α-terpineol	250 ^e^	[35]	840.90	550.31	363.15
trans-carveol	4000 ^w^	[36]	-	-	1.09
nerol	500 ^e^	[31]	24.07	128.31	25.88
citronellol	100 ^e^	[32]	-	-	45.20
geraniol	30 ^e^	[32]	208.42	1044.32	304.56
1-decanol	400 ^e^	[37]	4.43	3.67	3.81
perilla alcohol	1100 ^w^	[38]	<1	2.59	-
borneol	140 ^w^	[36]	252.19	117.16	-
ethyl butanoate	20 ^e^	[39]	6.13	1.97	8.29
isopentyl acetate	30 ^e^	[40]	63.83	12.55	86.05
ethyl caproate	100 ^e^	[31]	8.73	3.85	16.29
methyl benzoate	5.2 ^w^	[27]	-	117.21	-
ethyl benzoate	575 ^e^	[35]	3.09	2.28	-
ethyl octanoate	5 ^e^	[35]	-	-	1099.91
neryl acetate	42 ^w^	[41]	489.03	85.05	52.58
geranyl acetate	150 ^w^	[27]	54.01	5.37	52.61
ethyl caprate	510 ^e^	[42]	10.30	<1	6.19
ethyl laurate	500 ^e^	[31]	1.76	-	3.04
ethyl isobutyrate	15 ^e^	[35]	5.06	-	-
methyl octanoate	200 ^w^	[31]	1.72	-	-
ethyl dodecanoate	500 ^e^	[31]	5.74	-	-
d-camphor	1360 ^w^	[38]	3.11	4.72	-
d-carvone	160 ^w^	[43]	-	-	15.79
piperitone	680 ^w^	[44]	4.15	7.21	<1
octanal	50 ^e^	[34]	-	-	3.59
octanoic acid	500 ^e^	[35]	8.46	-	7.57
decanoic acid	1000 ^e^	[35]	1.34	-	1.19
1,4-cineole	1.1 ^e^	[11]	1861.02	1319.73	658.08
1,8-cineole	550 ^e^	[45]	-	14.52	-

“-” indicates that the compound was not detected in the cultivar. ^w^ = odor threshold in water, ^e^ = odor threshold in ethanol-water, ^a^ = odor threshold in apple juice.

## Data Availability

The raw data supporting the conclusions of this article will be made available by the authors on request.

## Supplementary Materials

The following supporting information can be downloaded at: https://www.mdpi.com/article/10.3390/foods15162922/s1, Table S1. Total soluble solid (TSS) and total acid (TA) content in the three lemon cultivar fruit; Table S2. TSS, alcohols, glucose and citric acid content in the three lemon cultivar-based fermented fruit wines; Table S3. Concentration of volatiles in the three lemon cultivar-based fermented fruit wines.
