# Supplementary material for: Analysis of Flavor Characteristics in Lemon-Fermented Fruit Wines from Three Distinct Varieties Using Odor Activity Values Combined with Multivariate Statistical Approaches
Source: Foods. 2026 Aug 20;15(16):2922. doi: 10.3390/foods15162922 (PMC13512514; doi:10.3390/foods15162922)
Supplement: Supplementary file 1 [file foods-15-02922-s001.zip › foods-4406840-supplementary.pdf]

SUPPLEMENTARY

Analysis of Flavor Characteristics in Lemon-Fermented Fruit Wines from Three Distinct Varieties Using Odor Activity Values Combined with Multivariate Statistical Approaches

Sen Huang [1,2,†], Zhihong Lu [1,2,†], Jiying Zhang [1], Yu Zhang [3], Zegang Li [4], Wanfu Yao [5] and Yujiao Cheng [1,2,6,*]

1 Citrus Research Institute, Southwest University, Chongqing 400712, China
2 National Citrus Engineering Research Center, Chongqing 400712, China
3 College of Food Science, Southwest University, Chongqing 400712, China
4 Pingshan County Fengshuo Agricultural Investment Co., Ltd., 645350, China
5 Yuxi Academy of Agricultural Sciences, 653100, China
6 Key Laboratory of Quality and Safety Control of Citrus Fruits, Ministry of Agriculture and Rural Affairs, Chongqing 400712, China
* Correspondence: yujiaocheng@swu.edu.cn
† These authors contributed equally to this work.

Table S1：Total soluble solid (TSS) and total acid (TA) content in the three lemon cultivar fruit

**Samples**	**TSS/brix**	**TA(g/L)**
SLFW	2.1±0.1^{a}	0.65±0.01^{a}
PLFW	1.8±0.1^{b}	3.18±0.02^{b}
ELFW	2.1±0.1^{a}	6.97±0.01^{c}

Table S2：TSS, alcohols, glucose and citric acid content in the three lemon cultivar-based fermented fruit wines

Samples	TSS/brix	Alcohols%(V/V)	Glucose (g/L)	citric acid (g/L)
SLFW	18.4±0.4[a]	1.7±0.2[a]	153±18[a]	1.52±0.02[a]
PLFW	16.4±0.1[b]	3.1±0.1[b]	110±6[b]	6.94±0.36[b]
ELFW	19.4±0.4[a]	0.4±0.1[c]	173±8[c]	9.04±0.31[c]

Table S3：Concentration of volatiles in the three lemon cultivar-based fermented fruit wines

No	Compounds	Concentration (μg/L)		
		ELFW	SLFW	PLFW
	**Hydrocarbons**			
1	α-thujene	219.70±21.09	353.83±39.22	0.00
2	α-pinene	12725.12±480.57	3171.84±21.46	1770.76±339.83
3	camphene	3212.41±70.03	496.79±423.47	0.00
4	β-pinene	26575.61±464.24	16114.31±3149.20	0.00
5	β-myrcene	20725.71±527.68	7723.29±1940.40	13812.24±2765.09
6	α-phellandrene	2845.11±5.80	1420.94±207.34	1573.35±85.15
7	4-carene	10338.31±490.40	1366.12±417.17	0.00
8	d-limonene	180524.74±753.88	93249.45±15300.22	150878.36±20803.96
9	β-ocimene	3287.11±1.09	1360.18±25.92	1764.35±92.45
10	γ-terpinene	66317.34±767.85	25404.74±5695.42	1204.48±81.11
11	terpinolene	20985.12±327.08	3338.81±507.84	851.03±86.74
12	p-cymenene	1143.72±42.24	842.36±142.99	1326.06±183.42
13	p-1,3,8-menthatriene	975.03±153.94	418.50± 29.88	765.38±116.12
14	trans-α-bergamotene	938.04±177.17	202.00±10.22	721.54±632.47
15	α-santalene	701.19±160.90	75.48±3.02	0.00
16	caryophyllene	7133.28±1329.10	1229.44±63.41	687.70±135.33
17	α-bergamotene	9542.45±2023.80	1931.81±31.52	1268.71±213.06
18	(Z)-β-farnesene	904.43±211.46	182.16± 8.84	118.21±19.47
19	humulene	1412.15±276.57	508.89±9.72	0.00
20	valencene	7676.17±1097.35	155.50±14.20	0.00
21	cis-α-bisabolene	0.00	0.00	129.87±11.39
22	eremophilene	8077.93±1495.75	0.00	0.00

23	(-)-β-bisabolene	12057.31±2264.42	2647.59±100.95	1519.03±201.80
24	(-)-β-pinene	12576.38±2038.95	2790.08±261.77	3463.21±139.34
25	isodurene	415.34±24.24	0.00	0.00
	**Alcohols**			
26	ethanol	24448.24±1627.63	14755.18±480.59	24222.62±1282.55
27	propanol	832.18±339.99	293.26±35.36	975.73±46.40
28	isobutanol	2971.48±529.62	964.02±184.34	3933.90±357.82
29	3-methylbutanol	22033.25±393.40	6944.66±1075.39	28915.73±1804.97
30	2-methyl-1-butanol	2441.88±465.62	1707.86±130.31	1261.09±148.73
31	3-hexen-1-ol	0.00	1019.04±55.61	0.00
32	1-hexanol	0.00	2044.32±130.07	0.00
33	1-octanol	4691.42±1015.95	8302.33± 240.57	3883.17±195.11
34	linalool	17452.20±2136.98	47260.68±1546.87	40440.15±2084.07
35	fenchol	44654.16±3310.50	21076.18±473.78	3213.68±209.08
36	phenylethyl alcohol	3729.54±315.35	7015.64±907.14	12951.94±1297.10
37	β-terpineol	5580.69±260.16	2418.53±374.43	19003.94±603.87
38	camphene hydrate	4511.16460.50	7872.10±964.74	0.00
39	4-terpineol	115855.27±12369.7	159796.47±7102.88	25704.50±620.23
40	α-terpineol	210225.72±6846.23	137578.70±4013.42	90787.51±1685.06
41	trans-carveol	0.00	0.00	4370.94±502.26
42	γ-isogeraniol	0.00	3060.79±92.01	0.00
43	nerol	12037.40±774.22	64153.28±1660.29	12938.66±2866.95
44	citronellol	0.00	0.00	4520.44± 299.09
45	cis-isogeraniol	0.00	4307.66±172.51	0.00
46	geraniol	6252.49±720.09	31329.72±1442.44	9136.70±125.31
47	1-decanol	1773.08±349.66	1469.27±91.74	1524.48±180.79
48	perilla alcohol	849.45±80.68	2846.85±307.94	0.00
49	α-bisabolol	220.58±72.90	453.37±0.61	196.13±4.97
50	2-methyl-3-buten-2-ol	227.11±8.54	384.73±21.88	70.92±1.96
51	(E)-3-hexen-1-ol	0.00	281.66± 14.17	0.00
52	(Z)-3-Hexen-1-ol	0.00	859.44±94.83	0.00
53	cis-p-2-menthen-1-ol	0.00	821.37±156.51	0.00
54	isopulegol	0.00	1958.17±206.23	7274.90±1583.21
55	isopregol	0.00	0.00	4187.77±870.18
56	borneol	35306.53±2860.25	16402.12±1472.41	0.00
	**Esters**			
57	ethyl acetate	2154.99±60.32	3030.14±188.24	1459.40±119.38
58	ethyl butanoate	122.53±10.24	39.47±2.47	165.79±21.30
59	isopentyl acetate	1915.03±215.56	376.62±41.83	2581.56± 157.92
60	ethyl caproate	873.02±1045.34	384.73±29.47	1628.68±169.98

61	methyl benzoate	0.00	609.52±70.63	0.00
62	ethyl benzoate	1776.93±430.57	1312.30±267.96	0.00
63	ethyl octanoate	0.00	0.00	5499.56±3553.15
64	ethyl nonanoate	2166.29±1072.21	0.00	0.00
65	nonyl acetate	731.56±179.43	0.00	0.00
66	neryl acetate	20539.16±520.32	3571.99±183.36	2208.32±214.39
67	geranyl acetate	8102.21±36.83	805.24±46.12	7891.43±342.79
68	ethyl 9-decenoate	1736.73±908.01	0.00	752.95±128.15
69	ethyl caprate	5253.00±139.11	317.39±18.73	3155.26±542.50
70	ethyl undecanoate	498.32±11.95	0.00	63.71±8.07
71	ethyl laurate	880.13±74.83	0.00	1520.33±9.64
72	ethyl isobutyrate	75.89±3.42	0.00	0.00
73	ethyl octanoate	11729.43±748.26	0.00	4423.06±735.36
74	ethyl dodecanoate	2870.76±349.88	0.00	0.00
	**Ketones**			
75	d-camphor	4225.05±46.27	6424.28±85.04	0.00
76	d-carvone	0.00	0.00	2525.72±189.00
77	piperitone	2821.72±171.69	4903.59±65.23	74.00±0.30
	**Aldehydes**			
78	acetaldehyde	1872.43±57.42	826.33±10.88	1073.74±90.77
79	octanal	0.00	0.00	179.57±8.40
	**Acids**			
80	acetic acid	1612.97±19.15	0.00	599.10±20.43
81	octanoic acid	4227.72±161.75	0.00	3787.18±277.70
82	decanoic acid	1337.41±27.18	0.00	1192.59±15.89
	**others**			
83	carvacrol	0.00	330.60±14.21	0.00
84	1,4-cineole	2047.12±48.10	1451.70±60.20	723.89±30.65
85	1,8-cineole	0.00	7985.43±175.06	0.00
